# Echocardiographic imaging of a bifurcated double barrel coronary sinus

**DOI:** 10.1186/s13019-023-02105-8

**Published:** 2023-01-19

**Authors:** Kendra L. Walsh, Andrew Winegarner, Geoffrey L. Hayward

**Affiliations:** 1 The Warren Alpert Medical School of Brown University, Providence, RI USA; 2 Department of Anesthesia at The Warren Alpert Medical School of Brown University, Providence, RI USA; 3 Pharmacy Services, Rhode Island Hospital, Providence, RI USA

**Keywords:** Double-barrel coronary sinus, Cardiothoracic surgery, Cardiothoracic anesthesia, Cardioplegia, Cardiac bypass, Transesophageal echocardiography

## Abstract

**Background:**

The coronary sinus (CS) is the terminal collecting vessel of the myocardial venous network, which returns deoxygenated blood used by the heart to the right atrium. The advent of high-fidelity imaging via CT and transesophageal echocardiography (TEE) has further defined the anatomy of the CS and its multiple tributaries. Understanding this anatomy is crucial for cardiac surgical cases that require the cannulation of the coronary sinus to deliver retrograde cardioplegia. However, anatomical variants of the CS may frustrate surgical retrograde catheter placement, in turn increasing the risk of CS injury or leading to inadequate cardioplegia delivery. Here, we present an especially unique CS presentation, a bifurcated, double-barrel CS, which was discovered via intraoperative TEE imaging that revealed a CS with two smaller lumens instead of the singular large os.

**Case presentation:**

A 67-year-old male presented for ascending aortic dissection repair, aortic valve replacement, and single vessel coronary artery bypass graft. On the pre-bypass TEE exam, the anesthesiologist noted a bifurcated CS with two small lumens. The surgeon utilized this information to select a smaller diameter retrograde catheter to avoid damage or perforation of the vessel. With TEE guidance, the surgeon successfully cannulated one of the CS lumens. However, it was noted upon dosing of retrograde cardioplegia that all tributary vessels attached to the non-cannulated lumen remained devoid of cardioplegia. The surgeon was forced to repeatedly administer anterograde cardioplegia via a handheld catheter through the coronary ostium throughout the case. The operative field was also flooded with topical ice saline slush to ensure cardiac protection. Ultimately, the operation was completed without incident despite the non-ideal conditions resulting from this anatomic variant.

**Conclusions:**

Discovery of this patient’s double-barrel CS during the pre-bypass TEE was incidental, showing that such anatomical variants may be completely asymptomatic and benign in the non-operative setting. However, the delivery of cardioplegia proved challenging for this patient, highlighting some degree of risk with certain cardiac interventions. This case demonstrates the utility of intraoperative TEE to quickly ascertain unforeseen anatomical variants of the CS which could compromise the safety of cardiac surgery cases.

## Background

The coronary sinus (CS) is the terminal collecting vessel of the myocardial venous network, which returns deoxygenated blood used by the heart to the right atrium. The advent of high-resolution CT and transesophageal echo (TEE) imaging has further defined the anatomy of the CS and its multiple tributaries including the great cardiac vein, middle cardiac vein, and the small cardiac vein [1]. Understanding this anatomy is crucial for cardiac surgical cases that require the cannulation of the coronary sinus such as retrograde cardioplegia, cardiac resynchronization therapy and so on. Anterograde and retrograde cardioplegia have differences in flow rate and mechanism, but a retrograde approach is often considered in situations such as concomitant aortic insufficiency or diffuse obstructive coronary artery disease [2, 3]. The CS is typically cannulated in a blind fashion with indirect TEE support to assist in visualizing the anatomy [3]. However, anatomical variants of the CS may frustrate surgical retrograde catheter placement, in turn increasing the risk of CS injury or leading to inadequate cardioplegia delivery. Here, we present an especially unique CS presentation, a bifurcated, double-barrel CS, which was discovered via intraoperative TEE imaging that revealed a CS with two smaller lumens instead of the singular large os.

## Case presentation

**Fig. 1:**
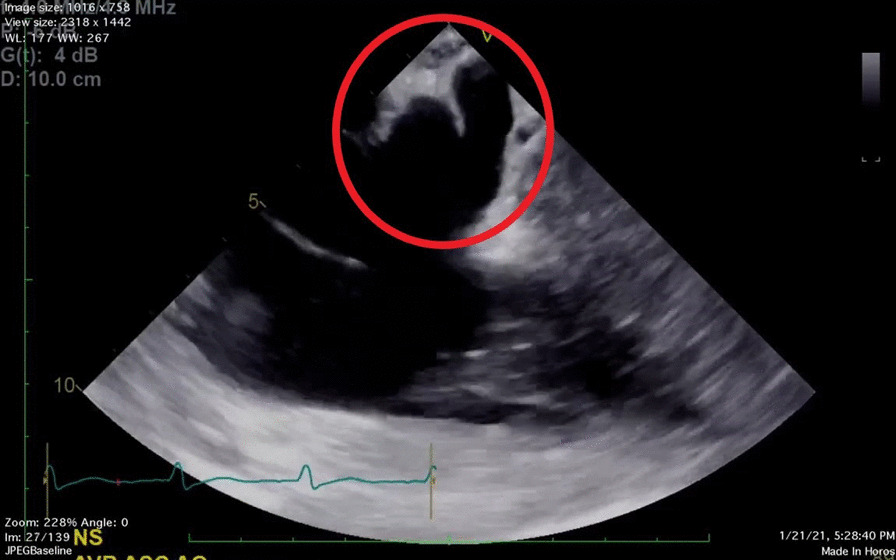
Lower Esophageal coronary sinus long axis view demonstrating a bifurcated coronary sinus within the circle

**Fig. 2:**
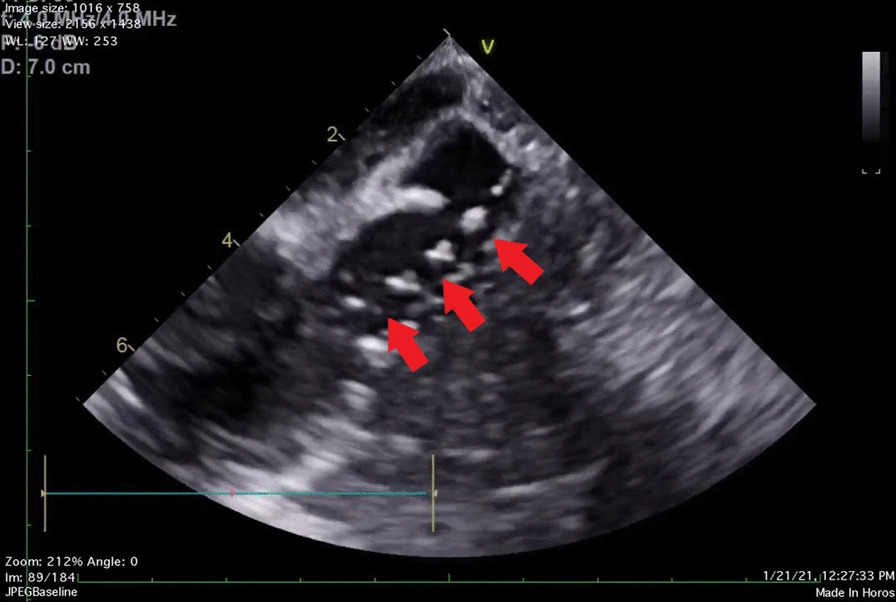
Lower Esophageal coronary sinus long axis view demonstrating the retrograde coronary sinus catheter placed within one of the bifurcated lumens. The acute angle of the second bifurcated sinus lumen to the catheter illustrates the anatomical hurdles to adequate retrograde cardioplegia

A 67-year-old male presented for the repair of a chronic type A ascending aortic dissection, aortic valve replacement, and single vessel coronary artery bypass graft revision. The patient’s past medical history included a prior CABGx3 in 2008 at an outside hospital, coronary artery disease, rheumatic aortic stenosis, hypertension, diabetes, and dyslipidemia. Of note, one of the patient’s bypass grafts (saphenous vein graft to the first diagonal artery) was occluded as it arose from the false lumen of the ascending aortic dissection. On the pre-bypass TEE exam, the anesthesiologist noted a bifurcated CS with two small lumens (approximately 0.4 cm and 0.5 cm in luminal diameter) (Fig. 1). The surgeon utilized this information to select a smaller diameter retrograde catheter to avoid damage or perforation of the vessel. With TEE guidance, the surgeon successfully cannulated one of the CS lumens (Fig. 2). However, it was noted upon dosing of retrograde cardioplegia that all tributary vessels attached to the non-cannulated lumen remained devoid of cardioplegia, suggesting the bifurcation was noncommunicating between the two lumens. As a result of this blockage, the surgeon was forced to repeatedly administer anterograde cardioplegia via a handheld catheter through the coronary ostium throughout the case. The operative field was also flooded with topical ice saline slush to ensure cardiac protection. Ultimately, the operation was completed without incident despite the non-ideal conditions resulting from this anatomic variant.

## Discussion and conclusions

Discovery of this patient’s double-barrel CS during the pre-bypass TEE was incidental showing that such anatomical variants may be completely asymptomatic and benign in the non-operative setting. In fact, this CS abnormality was not noted during this patient’s prior surgery in 2008. However, given the septation in his CS, the delivery of cardioplegia proved challenging for this patient. Embryologically, this unique finding is believed to result from the incomplete fusion of the left horn of the sinus venous with the adjacent great cardiac vein during the 10th week of fetal development. As such, this anatomic variant may be detected in people of all ages [4, 5]. Across the medical literature, approximately five case reports have been published on the double-barrel CS, proving it to be exceedingly rare. As this is a benign condition outside of the cardiac surgical patient population, it is likely highly underreported in otherwise healthy individuals [5–9].

This case demonstrates the utility of intraoperative TEE to quickly ascertain unforeseen anatomical variants of the CS which could compromise the safety of cardiac surgery cases. With the CS being a fragile structure, repeated attempts at cardioplegia cannulation could prove disastrous if the anatomy is found to be atypical. It has been reported that CS canulations are unsuccessful in 5–10% of patients undergoing invasive cardiac procedures. Anatomic variants such as prominent the besian valves being a large contributor to such frustrations [10]. Although closer in proximity to the inferior vena cava, the eustachian pouch may also appear on right atrial imaging and confound coronary sinus interrogation [11].

In this instance, TEE imaging allowed the surgical team to anticipate challenges with retrograde cardioplegia prior to the moment of acute need during the case. It also prompted preparations for contingency strategies including use of a smaller diameter CS catheter, handheld anterograde cardioplegia, and direct cooling of the surgical field with ice. One case report suggests that the decision on which lumen should be cannulated can be determined based on which part has greater flow on Doppler; this presumes it indicates the lumen with the greatest myocardial coverage, though there is no empirical evidence to support this notion [9]. Of course, the decision to use anterograde cardioplegia at baseline is another option, which can avoid potential inadequate coverage of the right heart seen when only retrograde cardioplegia is used, but this approach must take into consideration both the planned surgical procedure and also other patient specific factors [2].

Given the relative obscurity of this anatomy and its interesting clinical presentation, we believed it prudent to document its imaging and clinical course for future reference. For surgical cases requiring retrograde cardioplegia, TEE should be considered prior to bypass to confirm normal anatomy, or in our case, prepare for future challenges awaiting the surgeon. In such cases where the CS anatomy is dramatically altered, considering alternatives to retrograde cardioplegia is advisable.

## Data Availability

Not applicable.

## Abbreviations

CABG: Coronary artery bypass graft
CS: Coronary sinus
CT: Computed tomography
EKG: Electrocardiogram
PCI: Percutaneous coronary intervention
TEE: Transesophageal echocardiography
